# Exploring the potential of AI‐powered applications for clinical decision‐making in gynecologic oncology

**DOI:** 10.1002/ijgo.70251

**Published:** 2025-06-13

**Authors:** Bastian Meyer, Raphael Kfuri‐Rubens, Georg Schmidt, Maliha Tariq, Caroline Riedel, Florian Recker, Fabian Riedel, Marion Kiechle, Maximilian Riedel

**Affiliations:** ^1^ Department of Gynecology and Obstetrics TUM University Hospital, Technical University Munich Munich Germany; ^2^ Institute of Computational Biology, Helmholtz Zentrum München, German Research Center for Environmental Health Neuherberg Germany; ^3^ Department of Medicine III, Hematology and Oncology TUM University Hospital, Technical University Munich Munich Germany; ^4^ TUM School of Medicine Technical University of Munich Munich Germany; ^5^ Department of General Internal Medicine and Psychosomatics Heidelberg University Hospital Heidelberg Germany; ^6^ Department of Gynecology and Obstetrics Bonn University Hospital Bonn Germany; ^7^ Department of Gynecology and Obstetrics Heidelberg University Hospital Heidelberg Germany

**Keywords:** breast cancer, ChatGPT, clinical decision‐making, gynecology, large language model, tumor board

## Abstract

**Objective:**

The rise of artificial intelligence (AI) and large language models like Llama, Gemini, or Generative Pretraining Transformer (GPT) signals a promising new era in natural language processing and has significant potential for application in medical care. This study seeks to investigate the potential of GPT‐4 for automated therapy recommendations by examining individual patient health record data with a focus on gynecologic malignancies and breast cancer.

**Methods:**

We tasked GPT‐4 with generating independent treatment proposals for 60 randomly selected patient cases presented at gynecologic and senologic multidisciplinary tumor boards (MDTs). The treatment recommendations by GPT‐4 were compared with those of the MDTs using a novel clinical concordance score and were reviewed both qualitatively and quantitatively by experienced gynecologic oncologists.

**Results:**

GPT‐4 generated coherent therapeutic recommendations for all clinical cases. Overall, these recommendations were assessed by clinical experts as moderately sufficient for real‐word clinical application. Deficiencies in both accuracy and completeness were especially noted. Using a quantitative clinical concordance score, GPT‐4 consistently demonstrated superior performance in managing the senologic cases compared with the gynecologic cases. Iterative prompting substantially enhanced treatment recommendations in both categories, increasing concordance with MDT decisions to up to 84% in senologic cases.

**Conclusion:**

GPT‐4 is capable of processing complex patient cases and generates detailed treatment recommendations; however, differences persist in surgical approaches and the use of systemic therapies, and there is a tendency toward recommending excessive genetic testing. As AI‐powered solutions continue to be integrated into medicine, we envision the potential for automated therapy recommendations to play a supportive role in human clinical decision‐making in the future.

## 1 INTRODUCTION

The Generative Pre‐Trained Transformer 4 (GPT‐4) represents a cutting‐edge AI tool within the realm of large language models (LLMs). GPT‐4 generates its responses by using deep learning algorithms trained on extensive text datasets.^1^ This training enables the model to understand the structure, syntax, and semantics of natural language.^2^ The model selects the most likely next token from a large set of sub‐word token embeddings, thus mimicking human language and providing relevant information or assistance.^3^ Since its release, GPT has been used extensively to respond to medical queries. Remarkably, it has been successfully tested on original questions from both American and Japanese medical exams demonstrating performances indicative of a passing grade.^4^, ^5^ In contrast, its responses to Korean medical exams were less accurate.^6^ Further examination revealed that GPT's performance tends to decline as the complexity and difficulty of the questions increase.^7^

In this project, we aimed to assess GPT‐4's ability to make diagnostic and therapeutic decisions using real‐world data from multidisciplinary tumor boards (MDTs) for gynecologic and senologic cases. Unlike other commercial AI‐based decision support systems, GPT‐4 offers free access and a user‐friendly interface, lowering the barrier to practical implementation. Building on the existing literature and our own data demonstrating GPT's success in obstetrics and gynecology (OB/GYN) exams, we hypothesized that GPT‐4 could handle complex oncologic cases and provide medically accurate, guideline‐compliant treatment recommendations.

Our study aimed to address and discuss three core questions:

1. How effectively does GPT‐4 perform to provide treatment recommendations for complex gynecologic and senologic MDT cases?
2. What is the level of concordance between GPT‐4's treatment recommendations and those of the MDTs, and how can this concordance be improved?
3. What are the potential applications and limitations of GPT‐4 in clinical and oncological decision‐making?

## 2 METHODS

### 2.1 Data acquisition and processing

In all, 30 gynecologic and 30 senologic primary patient cases were randomly selected from MDT meetings between April 2023 und April 2024 at the department of obstetrics and gynecology at the TUM University Hospital of the Technical University Munich (Germany). Ethics approval was obtained in advance from the ethics committee of TU Munich (ref: 2023‐517‐S‐SB). No informed consent could be obtained due to the retrospective nature of the study. In retrospect, 77% (*n* = 23/30) of the gynecologic cases and 90% (*n* = 27/30) of the senologic cases involved presentations concerning malignancy or suspected malignancy (Figure S1). To standardize the prompts for GPT‐4, the information from the MDTs was collected and attributed to the following categories: age, medical history, tumor histology and biology, clinical examination, and imaging. The German language and medical terminology were exclusively used throughout data acquisition and processing. No modifications were made to the original information from the MDTs; thus, abbreviations, spelling errors, and ambiguities were left uncorrected to simulate an authentic setting. Anonymized patient data from the cases were provided to GPT‐4, which was then tasked with generating its own treatment recommendations.

### 2.2 Data analysis

The original MDT recommendations for each patient were broken down into individual items. An item corresponded to a proposed intervention, for example, hysterectomy, initiation of chemotherapy, or genetic testing. For instance, if the MDT recommended a hysterectomy and genetic testing for a patient, these interventions were counted as two separate items. Additionally, if lymph node sampling was included in the recommendation, this intervention was counted as another separate item for analysis. Thus, we ensured that each distinct intervention was considered individually. Each patient case was assigned a maximum number of items achievable, thus establishing a clear framework for structuring natural language predictions. This allowed for a precise evaluation of the comprehensiveness and accuracy of the treatment recommendations generated by GPT‐4.

GPT‐4's therapy recommendations were evaluated for concordance with MDT decisions by analyzing both the quantity and quality of occurrence of each item within the treatment recommendations. This assessment involved categorizing each item as: “not mentioned” (0 point), “partially mentioned” (0.5 points) and “fully mentioned” (1 point). For example, an item was considered “partially mentioned” if a lymph node procedure was recommended, but GPT suggested an axillary lymph node dissection (ALND) instead of the original recommendation for a sentinel lymph node biopsy (SLNB).

To quantify this alignment, we established and calculated the clinical concordance score (CCS). This score was derived by computing the arithmetic mean of the number of item occurrences relative to the total number of items identified by MDTs for each patient case.

In detail, let $s_{\mathrm{CCS}}$ be the clinical concordance score for a specific patient case, defined as:

$$s_{\mathrm{CCS}}=\frac{1}{m}\sum_{j=1}^{m}I\left(o_{j},i_{j}\right)$$

where m is the total number of items identified by the MDT for this patient case, and $o_{j}$ is the observed occurrence of an item that corresponds to the MDT‐identified item $i_{j}$. The indicator function $I\left(o_{j},i_{j}\right)$ assigns a score based on the level of matching between *o*_*j*_ and *i*_*j*_, and is defined as:

$$\begin{array}{c} I\left(o_{i},i_{j}\right)\left\{\begin{array}{c} 1\;\text{if}\;o_{j}\;\text{fully matches the}\;\mathrm{MDT}-\text{identified item}\;i_{j}\;\\ 0,5\;\text{if}\;o_{j}\;\text{partially matches the}\;\mathrm{MDT}-\text{identified item}\;i_{j}\\ 0,\text{if}\;o_{j}\;\text{does not matches the}\;\mathrm{MDT}-\text{identified item}\;i_{j} \end{array}\;\right. \end{array}$$

In this formulation, $o_{j}$ represents the observed occurrence of each item j, corresponding to the MDT‐identified item $i_{j}$, and the summation is taken over all *m* items identified by the MDT for the patient case. Lastly, the subscript CCS1 in $s_{\mathrm{CCS}1}$ denotes the score from the initial assessment, while CCS2 represents the score after re‐prompting.

This quotient provides an approximation of how closely GPT‐4's recommendations match the treatment strategy developed by the MDT. Consequently, the CCS serves as a quantitative indicator of the degree of alignment between GPT‐4's generated recommendations and the established expert‐derived therapeutic interventions from the MDT.

**FIGURE 1:**
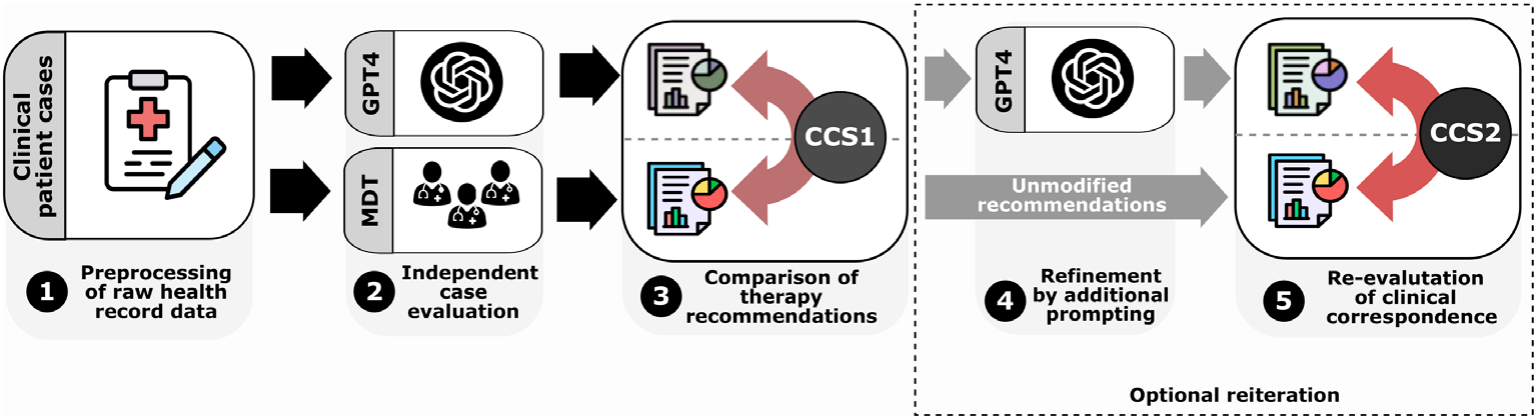
Schematic representation of data acquisition: Patient cases were discussed in multidisciplinary tumor board (MDT) meetings and were additionally provided to GPT‐4 to generate therapy recommendations. The therapy recommendations from the MDTs were compared with those generated by the large language model by calculating a clinical concordance score (CCS1). In a selection of cases, further interaction with GPT‐4 was facilitated through follow‐up questions, resulting in a second, revised set of therapy recommendations which was analyzed accordingly (CCS2).

In a subsequent prompt, further interaction with GPT‐4 was facilitated using simple, open‐ended follow‐up questions, leading to improved responses in both senologic (47%, *n* = 14/30) and gynecologic cases (63%, *n* = 19/30). These questions included, for example, “Should systemic therapy also be administered?” or “Should lymph nodes also be examined?”. As a result, a second set of therapy recommendations was generated and analyzed accordingly. This approach aimed at simulating an assistant‐like, realistic interaction with the AI model in the clinical setting (see Figure 1 for illustration of the workflow).

We then applied a two‐step approach for qualitative data analysis. First, patient cases and therapy recommendations generated by GPT‐4 were presented to three clinical experts specializing in gynecologic oncology and breast cancer. They evaluated GPT‐4's recommendation by using a five‐point Likert scale, in which 1 indicated “very poor” and 5 indicated “very accurate”, thus providing a global assessment of the AI‐generated recommendations.

Second, we assessed GPT‐4's responses using five key dimensions of response quality, originally defined by Richard Wang and Diane Strong.^8^ These dimensions have been frequently used to assess reliability, accuracy, and utility of data acquisition. By applying these pre‐defined categories, the evaluation of GPT's responses aligns with established data quality principles and offers a structured and comprehensive approach to assess the quality of AI‐generated information. Moreover, it has been previously used by us to evaluate GPT's performance on solving medical exam questions.^7^

We differentiated the following five dimensions of data acquisition:

1. *Ease of understanding:* Was the answer clearly and precisely formulated in a way that was easy to understand?
2. *Concise representation:* Was the answer clearly structured and divided into sections that facilitated readability?
3. *Accuracy:* Did the facts mentioned in the answer correspond to the current scientific literature? Were the statements logical and understandable?
4. *Completeness:* Was the answer complete, and were all aspects of the question adequately addressed? Was important information omitted, or were there unnecessary details?
5. *Relevance:* Was the answer directly related to the question asked, or was there any ambiguity in the answer?

For this analysis, three experienced physicians in the field of gynecologic oncology assessed the answers by GPT‐4 independently with regard to the five items mentioned in the list using a five‐point Likert scale (ranging from 1 = “completely disagree” to 5 = “completely agree”).

### 2.3 Statistical analysis

The data were evaluated using Excel (version 16.78, 2023, Microsoft) or Prism (version 10.3.1, 2024, GraphPad). Unpaired *t*‐test was used to compare the expert assessment of GPT‐4's responses via Likert scale ratings. Paired *t*‐test was used to compare GPT‐4's first and revised therapy recommendations. All *P*‐values <0.05 were defined as statistically significant. Tables and figures were generated in Excel, Prism, or Adobe Illustrator (version 28.7.1, 2024, Adobe). Mathematical functions and equations were typeset in LaTeX (version 4.8.3, 2024, LaTeX Project Team).

## 3 RESULTS

### 3.1 GPT‐4 delivered therapy recommendations for all gynecologic and senologic cases

For all of the 60 gynecologic and senologic cases, GPT‐4 was able to provide therapeutic recommendations. These recommendations by GPT‐4 had a mean word count of 38.6 (standard deviation [SD] 10.7) for the gynecologic and 50.9 (SD 32.9) for the senologic cases, compared with 10.2 words (SD 6.3) and 18.9 words (9.8) in the original MDT, respectively. Tables S1 and S2 display the items attributed to each patient case, highlighting which of them were mentioned by GPT‐4 in the initial or revised response.

### 3.2 Mixed review of the quality of the AI‐generated treatment recommendations

**FIGURE 2:**
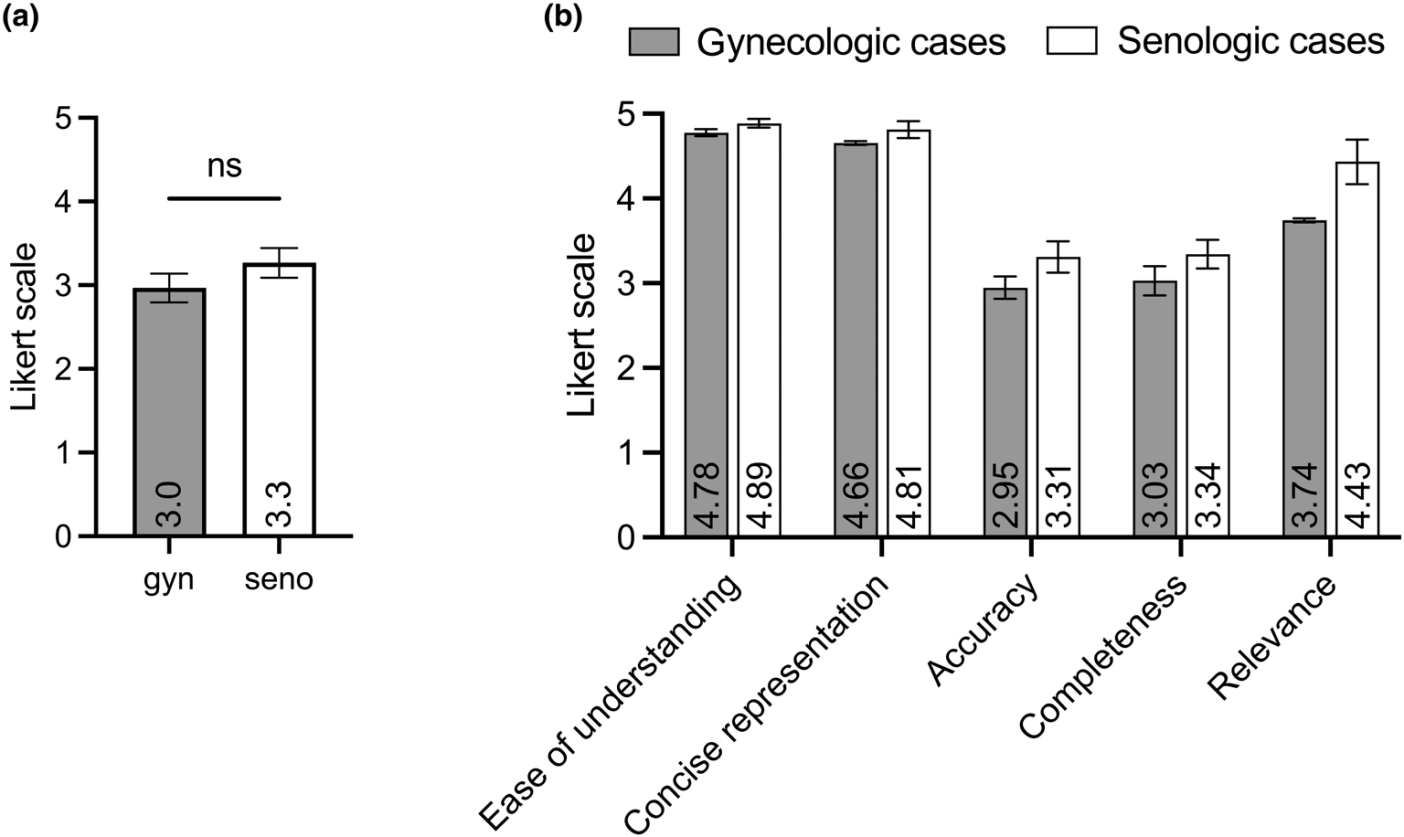
(a) Evaluation of AI‐generated therapy recommendations for gynecologic (gyn) and senologic (seno) cases by experienced physicians. The mean value on a Likert scale (1 = “very poor” to 5 = “very good”) and the standard deviation (ns = not significant) are provided. (b) Qualitative evaluation of GPT‐4's treatment recommendations based on the established five data quality categories. The mean values and standard deviations of the ratings for each category, as evaluated by three experienced oncologists, are presented.

Regarding the expert overall opinion on the quality of GPT‐4's therapy recommendations, the AI‐generated suggestions were considered moderately sufficient for real‐world clinical application. Specifically, the gynecologic therapy recommendations received an average rating of 3.0 on the five‐point Likert scale, while the senologic cases were rated slightly higher with an average of 3.3 (1 indicating “very poor” and 5 indicating “very accurate”). However, this difference was not statistically significant (Figure 2a). With regard to the key dimensions of data quality defined by Wang and Strong,^8^ GPT‐4 performed best in the categories of “ease of understanding” and “concise representation.” The category “relevance,” which assesses whether the answer directly addresses the question, also received high ratings. In contrast, “accuracy” and “completeness,” which evaluate conformity with current treatment standards and consideration of all available information, received lower ratings (Figure 2b).

### 3.3 GPT demonstrated better performance in senologic cases

**FIGURE 3:**
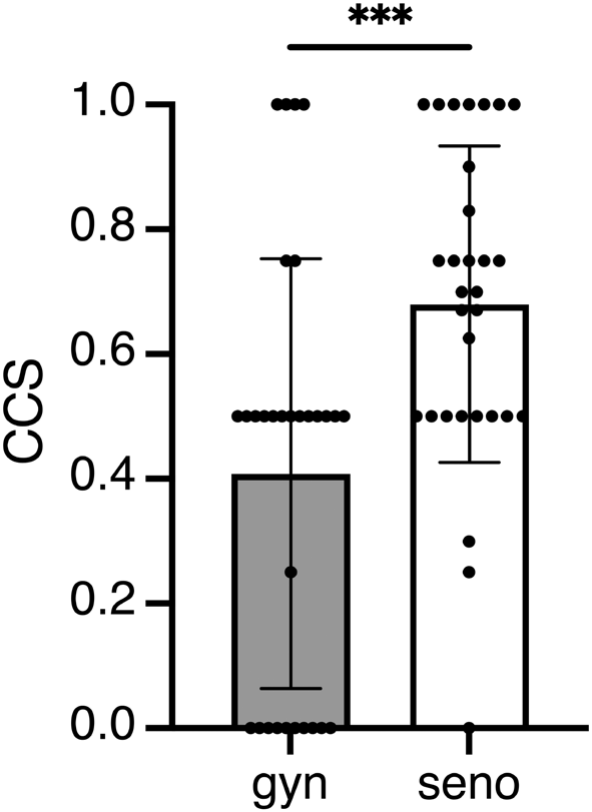
The clinical concordance score (CCS) for the initial therapy recommendations from GPT‐4 is a ratio representing the agreement between the AI's therapy suggestions and those of the tumor board. The CCS ranges from 1 (complete agreement, with all items matched) to 0 (no agreement in therapy recommendations). Individual values per patient are depicted as dots along with the mean and standard deviation for gynecologic (gyn) and senologic (seno) patient cases. Unpaired t‐test was used for statistical analysis (***P < 0.001).

We identified 75 and 45 individual items across all senologic (mean per case = 2.5, SD 1.28) and gynecologic patient cases (mean per case = 1.5, SD 0.57), respectively. In comparison, GPT‐4 achieved a total of 52 items (mean per case = 1.7, SD 1.1) in the senologic cases and 19.5 items (mean per case = 0.65, SD 0.65) in the gynecologic cases. We further applied the aforementioned CCS; a CCS of 1 indicates that all items for a patient were addressed in the AI‐generated therapy recommendations, whereas a CCS of 0 indicates that none of the items were addressed. The mean CCS for the gynecologic patient cases was 0.41 (SD 0.34), whereas for senologic cases, it was significantly higher at 0.68 (SD 0.25) (*P* < 0.001) (Figure 3).

### 3.4 Additional interaction with GPT‐4 significantly improves its performance

**FIGURE 4:**
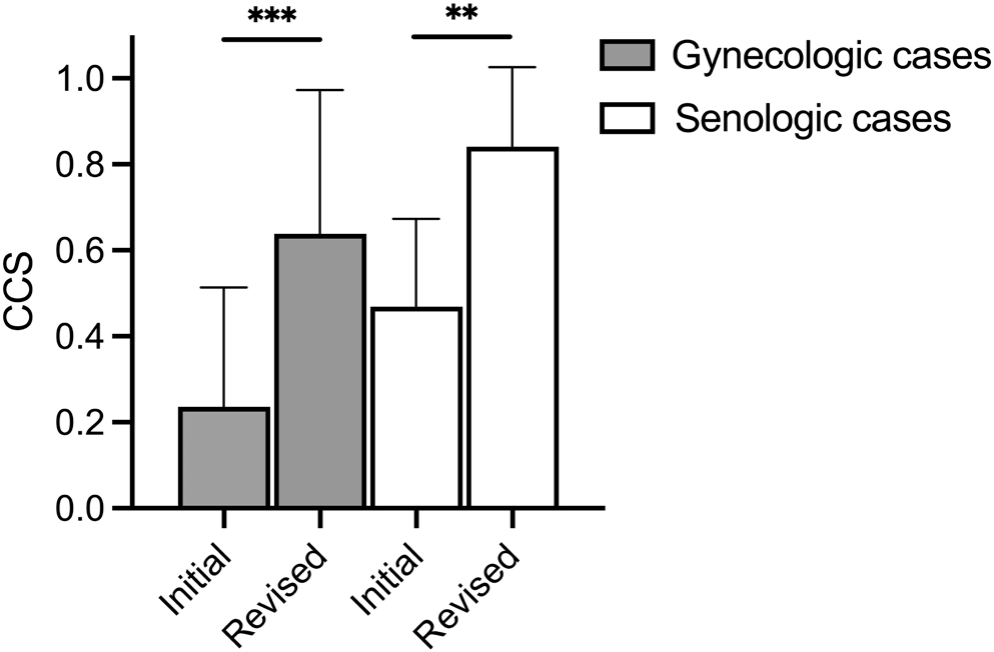
Changes in the clinical concordance score (CCS) after interaction with GPT‐4. For a subset of patient cases, simple follow‐up questions were posed after evaluating the initial treatment recommendation. This led to a second, revised treatment recommendation from GPT‐4. The graph displays the mean and standard deviation of the CCS for the initial and revised responses for gynecologic and senologic cases. Paired *t*‐test was used for statistical analysis (**P < 0.01, ***P < 0.001).

In a randomly chosen subset of the gynecologic (*n* = 18) and senologic (*n* = 12) patient cases, the inherent function of the LLM was utilized to pose additional open questions, in order to optimize the initially suggested recommendations. This generated a second, revised response from GPT‐4 which was also evaluated for its agreement with the recommendations of the MDT by calculating the CCS. The CCS of treatment recommendations improved after the follow‐up questions were asked. For the gynecologic and senologic cases, the CCS increased from 0.24 (SD 0.27) to 0.64 (SD 0.33), and from 0.47 (SD 0.2) to 0.84 (SD 0.18), respectively. The revised, second treatment recommendations by GPT‐4 after the follow‐up questions received significantly higher ratings in both gynecologic and senologic cases (Figure 4).

## 4 DISCUSSION

To the best of our knowledge, this study represents the largest collection of MDT cases analyzed by an LLM in the field of gynecologic oncology and breast cancer.^9^, ^10^ Additionally, due to the minimal processing of patient data, this represents one of the first studies to be conducted in a realistic, real‐world clinical setting. Our results help to broaden the perspective on the performance of LLMs in the medical context. While numerous publications have demonstrated GPT's strong performances in answering theoretical medical questions—including fictional patient cases or guideline adherence—our project focused on its real‐world applicability, where a more nuanced picture of its potential emerges. The results of this study demonstrate GPT's capability to handle complex patient cases and provide specific treatment recommendations based on clinical information. Our data support existing studies that highlight GPT's strong medical understanding, demonstrated by its ability to pass medical licensing exams, such as the United States Medical Licensing Examination (USMLE) or the General Medicine In‐Training Examination (GM‐ITE), in part even outperforming medical students and residents.^4^, ^5^ Interestingly, particularly strong results were observed in questions related to gynecology.^5^ This aligns with our own data showing that GPT achieved a “passing grade” in our OB/GYN course and the national state exams comparable to that of final‐year medial students.^7^

In the present study, GPT‐4's responses showed the highest scores in the qualities of “ease of understanding”, “conciseness”, and “relevance”, but deficiencies in “accuracy” and “completeness.” Concordance between GPT‐4's treatment recommendations and those of the MDT improved with further interaction and the posing of follow‐up questions, reaching up to 84% for the senologic cases. We attribute the better results seen in the senologic cases versus the gynecologic cases to the nature of primary, non‐metastatic breast cancer, for which the clinical algorithms (imaging, surgery, adjuvant/neoadjuvant therapy, radiotherapy, etc.) are more straightforward and generally less ambiguous. This helps GPT to achieve good clinical reasoning. Moreover, our results surpass previous studies on this topic, for example by Lukac et al., who first compared GPT's therapy recommendations for senologic patients with those of MDTs and reported a 16.05% concordance.^11^ However, the results are not directly comparable. Our study used the updated version GPT‐4, which has addressed issues like hallucinations and performed better in benchmarks than GPT‐3.5.^12^, ^13^ This may explain why neoadjuvant therapies were suggested in our project, unlike in the study by Lukac et al. and using GPT‐3.5. Additionally, differences in prompts and patient information may further limit direct comparison between these studies.

Our study indicates that persistent errors—such as omissions and hallucinations—contribute to a mediocre evaluation of GPT's recommendations by expert physicians. This finding aligns with a recent study by Braun et al., which assessed GPT's responses to gyneco‐oncologic questions for guideline adherence, as evaluated by experts in palliative care and gynecologic oncology. They similarly concluded that the assessment of individual therapies, particularly filtering out incorrect recommendations, still requires the indispensable expertise of a medical professional.^14^

Effective prompting has a significant impact on the function of LLMs like GPT‐4. Research has shown that the use of structured and standardized prompts can lead to more accurate and relevant responses from the models.^15^, ^16^ As a result, the field of “prompt engineering” has emerged, leading to the development of optimized prompt recommendations specifically tailored to the medical use of LLMs, which we have followed in our approach.^15^, ^17^, ^18^ In addition to GPT‐4, several other LLMs have been evaluated for medical applications. In comparison, GPT‐4 outperformed both earlier versions of itself and other LLMs, for example, Llama2 and Google's Bard. Griewing et al. evaluated different versions of GPT and found that GPT‐4 demonstrated the highest overall concordance with human experts in breast cancer treatment recommendations. It achieved full concordance with radiotherapy recommendations and showed significant accuracy with regard to endocrine treatment and genetic testing. In contrast, earlier versions of GPT, as well as other models like Llama2 and Bard, displayed lower concordance rates, highlighting the advancements and improvements in the latest updates of GPT.^19^ This superior performance can be attributed to the advanced algorithms and extensive datasets used in training GPT‐4, which enhance its ability to comprehend and replicate complex medical reasoning.^20^

Our study demonstrates the feasibility of using GPT‐4 to generate treatment recommendations for complex gynecologic patients. It helps to expand the current understanding of the capabilities of LLMs by addressing specific clinical questions. The overarching goal is to explore practical applications of AI in routine clinical practice—with the potential to improve care for both healthcare professionals and patients. Several studies have already highlighted the potential of LLMs in patient education, such as answering individual patient questions,^21^ improving educational materials,^22^ and translating medical information into more accessible language for lay audiences.^23^ Evidence suggests that some physicians derive greater benefit from AI support than others. For instance, in the domain of skin cancer, less experienced clinicians have demonstrated greater improvement in decision‐making when assisted by AI compared with their more experienced colleagues.^24^ This highlights the potential use of LLMs in the education and training of early‐career physicians.

Furthermore, general practitioners may find the system especially helpful in managing diagnostic uncertainty or assessing complex information—especially in primary care settings where time is limited or specialist access is constrained. Nonetheless, their clinical judgment and supervision remain essential to ensure safe and effective use. The system is not intended to replace a medical consultation, nor should it be used to make treatment decisions without proper clinical validation. From the patient's perspective, LLMs can support shared decision‐making by helping individuals better understand clinical recommendations through clear, accessible explanations. By addressing unanswered questions and simplifying complex medical information, LLMs could reduce confusion, encourage informed participation in medical decisions, and ultimately improve adherence to prescribed therapies. Furthermore, LLMs offer the potential for a low‐threshold digital second opinion, empowering patients to seek additional guidance and reassurance outside of traditional consultations, especially when access to specialists is limited.

A particularly promising approach for clinical integration is the concept of human‐in‐the‐loop AI. In this model, the AI generates a recommendation or decision proposal, which is subsequently reviewed, adjusted, or validated by a healthcare professional. The final responsibility for the decision remains with the human expert.^25^ As physicians are familiar with the patients' individual life circumstances and personal backgrounds, it is essential to clearly distinguish between a purely formal treatment recommendation and the personalized treatment decision. Within the context of MDTs, automated screening for clinical trial eligibility represents another promising use case. Beattie et al. showed that LLMs exhibit strong capabilities in identifying eligible patients for clinical trials, reducing the workload of medical personnel and enhancing the efficiency of clinical trial recruitment.^26^

A limitation of our study is that the CCS has been applied for the first time. While it offers benefits like objectivity, comparability, and simplicity, our analysis only considered items mentioned by the MDT, and GPT‐4's recommendations beyond them—whether correct or incorrect—are excluded. This exclusion may explain discrepancies between the CCS and evaluations by gynecologic oncologists, who assessed the full therapeutic recommendation. Additionally, a “partially mentioned” item scored at 0.5 points can still significantly impact patient care, such as recommending sentinel lymph node biopsy when guidelines call for axillary lymph node dissection in advanced breast cancer. In addition, the sample size of 2 × 30 cases from the fields of senology and gynecology represents a limitation of the present study and does not allow for a definitive assessment of GPT‐4's performance in addressing gynecologic oncology‐related questions. Further research with a larger number of cases is necessary to account for the substantial heterogeneity in tumor biology as well as the individual variability within patient populations.

## 5 CONCLUSION

Our findings suggest that—with further improvements—GPT may become a valuable tool for clinicians treating complex oncologic patients. However, further research is necessary to refine the algorithms and improve the accuracy and completeness of the AI‐generated recommendations. This is especially relevant in the intricate medical field of oncology. As AI technologies continue to evolve, integrating systems like GPT‐4 into clinical practice offers considerable potential to improve everyday care—for both medical professionals and their patients. However, questions surrounding data security and patient privacy remain unresolved and continue to be the subject of ongoing debate.

## AUTHOR CONTRIBUTIONS

BM and MR designed the project, the main conceptual ideas, and the proof outline. FRe, FRi, and MK supervised the project. BM, GS, MT, CR, and MR were responsible for data collection and management. BM and MR analyzed the data and performed the analytic calculations. RKR verified the analytical methods. BM and MR took the lead in writing the manuscript. All authors provided critical feedback and helped shape the research and analysis. All authors discussed the results and contributed to the final manuscript.

## FUNDING INFORMATION

None.

## CONFLICT OF INTEREST STATEMENT

The authors have no conflicts of interest.

## Supporting information


Data S1.

## Data Availability

Data are available on request due to privacy/ethical restrictions. Contact maximilian.riedel@mri.tum.de.
