# Supplementary material for: Exploring the potential of AI‐powered applications for clinical decision‐making in gynecologic oncology
Source: Int J Gynaecol Obstet. 2025 Jun 13;171(2):698–704. doi: 10.1002/ijgo.70251 (PMC12553098; doi:10.1002/ijgo.70251)
Supplement: Supplementary file 1 — Data S1. [file IJGO-171-698-s001.docx]

**Supplementary files**

**
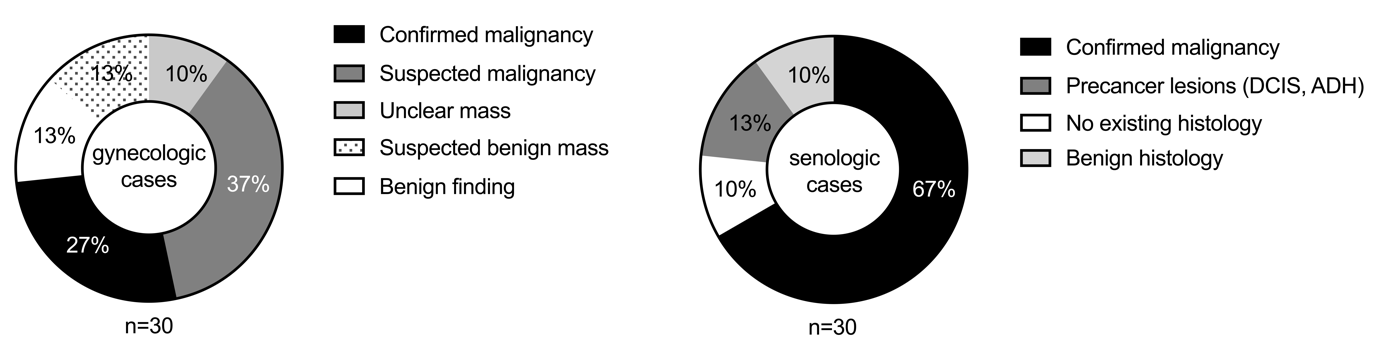
**

**Supplemental figure 1:** Distribution of present or suspected tumor entities in the MDTs

**Supplementary table 1:** **Senologic multidisciplinary tumor board (MDT)**

List of the therapy recommendations for each patient depicted as individual items proposed during the MDT. Each item is marked to indicate whether it was "fully mentioned" (1) or “partially mentioned” (0.5) by GPT-4 (GPT) in the respective category. The evaluation of the AI's response after further inquiry is shown in parentheses. The resulting clinical concordance score (CCS) is provided, along with the corresponding score after further inquiry (CCS2).

**Supplementary table 2: Gynecologic multidisciplinary tumor board (MDT)**

List of the therapy recommendations for each patient depicted as individual items proposed during the MDT. Each item is marked to indicate whether it was "fully mentioned" (1) or “partially mentioned” (0.5) by GPT-4 (GPT) in the respective category. The evaluation of the AI's response after further inquiry is shown in parentheses. The resulting clinical concordance score (CCS) is provided, along with the corresponding score after further inquiry (CCS2).
